# Preparing for the Next Potential Pandemic—Chikungunya, Dengue, Zika and Other Viruses

**DOI:** 10.3390/v18010037

**Published:** 2025-12-25

**Authors:** Zoltan Vajo, Csaba Laszlofy

**Affiliations:** 1 Department of Family Medicine, Semmelweis University Medical School, 1085 Budapest, Hungary; 2 Faculty of Dentistry, University of Szeged, 6720 Szeged, Hungary

## 1. Introduction

Pandemics have claimed millions of lives over millennia. The bubonic plague, smallpox, cholera, and, more recently, the Spanish flu devastated the world, resulting in more deaths than wars and enormous economic impacts. With the rapid advancement of medical knowledge during the 20th century, especially concerning infectious diseases and microbiology, it may appear that the importance of pandemics and the damage they cause will be much less concerning in the future. However, the massive threat of bird flu posed by Influenza A H5N1 and other highly pathogenic avian influenza viruses beginning in 2006, the subsequent emergence of swine flu in 2009–2010, the appearance of SARS and MERS, and, most importantly, the COVID-19 pandemic serve as wake-up calls, indicating that even with great advances in medicine, some aspects of our modern lifestyle, such as urbanization, massive sports events and concerts (with tens of thousands of people concentrating in small areas), indoor malls, and travel habits may actually increase the likelihood of future pandemics, reducing the effects of the epidemiological advances we have made.

Newly emerging or mutating variants of previously known microorganisms may continue to present potential for further pandemics. This Special Issue focuses on such viruses, including chikungunya, dengue, Zika, and others with pandemic potential.

## 2. Chikungunya

The Chikungunya virus is classified as an arbovirus, part of the Togaviridae family, with mosquitoes being its most important vector [1]. The most common symptoms of the infection are fever, muscle and joint pain, and rashes. Most people recover spontaneously, but in 30–40% of the affected patients, infection can lead to subacute joint inflammation, lasting weeks or more.

Thus far, at least 317,000 Chikungunya infections, with 135 Chikungunya-associated fatalities, have been confirmed across several separate geographic areas. Infections have been confirmed in all continents except Australia [2]. The ongoing appearance of Chikungunya viral infections has demonstrated that despite all measures taken so far to manage Chikungunya infections, it remains a significant concern not only in warmer climates but also in other parts of the world.

There are no specific antiviral treatment options available for chikungunya virus infection, making managing the threat posed by this virus even more challenging. Vaccines against the chikungunya virus, including live-attenuated and virus-like particle vaccines, are under development and being subjected to intense testing [3,4]. Two chikungunya virus vaccines have been licensed in the United States, namely, a live-attenuated vaccine (IXCHIQ) and a virus-like particle vaccine (VIMKUNYA), with the former being currently suspended due to safety concerns, especially for elderly patients [5]. This context further highlights the challenges we face with the next potential pandemic.

## 3. Dengue

Dengue fever is an acute febrile illness caused by one of the four serotypes of Dengue virus. It has variable clinical manifestations, ranging from asymptomatic to severe dengue hemorrhagic fever and shock. Extended and unusual presentations involving the heart, liver, kidneys, muscles, and brain have also been reported [6]. The Dengue virus is a flavivirus with a single-stranded RNA genome and comprises three structural proteins, a capsid, an envelope, and a membrane. The global incidence of dengue has recently increased significantly, with almost half of the world’s population now potentially at risk of infection [7]. Since there is no antiviral medication specifically for treating dengue fever, treatment options are severely limited, mainly constituting symptom relief and support. Infection with one serotype of the virus does not guarantee protection from the other serotypes, and reinfection with a different serotype may result in more severe symptoms [7,8]. Thus, the development of a safe tetravalent vaccine that produces a balanced immune response to all four serotypes has been a longstanding goal, and the most promising approach involves live attenuated virus vaccines, which represent their own significant challenges. Besides vaccination, effective dengue control also involves vector control, educational programs, and epidemiological surveillance [9].

## 4. Zika

The Zika virus encompasses a positive single-stranded RNA genome and belongs to the flaviviridae family [10]. The virus was first isolated in Uganda in 1947. It is considered an arbovirus transmitted primarily by mosquitoes. However, at least in some cases, person-to-person spread has also been verified [11,12]. Most Zika virus infections are asymptomatic or cause only mild, general symptoms such as the common cold. Frequent symptoms include a low-grade fever, malaise, myalgias, skin rashes, headaches, and joint pain, most commonly in the hands and feet. Importantly, Zika virus infections during pregnancy can cause serious complications, including microcephaly, along with Guillain–Barré syndrome in adults [13].

Despite recognition of the threat it poses, decades after its discovery, no vaccines have been granted approval against Zika virus, making the population globally vulnerable to potential future outbreaks [14]. Nonetheless, developments in several different pathways have recently been made to induce immunity against the Zika virus, and, together with the traditional inactivated and live-attenuated viral vaccines and the more recent DNA vector, mRNA-based, and recombinant protein vaccines, multiple platforms are moving towards randomized, controlled human trials.

## 5. Highly Pathogenic Avian Influenza (HPAI)

Highly pathogenic avian influenza viruses, mostly influenza A H5N1 and H7N9 have been considered a potential pandemic threat since the early 2000s. These strains cause severe disease with high fatality rates when transmitted from birds to humans, with a case/fatality ratio of up to 59% [15]. Human-to-human transmission has occurred, but it is infrequent [16]. The WHO set level 3/6 pandemic alertness in 2006 and recommended developing and stockpiling vaccines [17].

HPAI poses some unique challenges. Conventional diagnostic assays for detecting influenza infection and/or verifying protection, whether natural or vaccine-induced, have been found to show extreme intra- and interlaboratory variability. The traditionally used chicken-red-blood-cell based hemagglutination inhibition assays have been found to be insensitive and unreliable; therefore, the use of horse erythrocytes has been proposed to assess response to infection and/or vaccination [18]. Due to the information above, the World Health Organization (WHO) established H5 reference laboratories as part of the WHO Global Influenza Surveillance and Response System [19]. An additional challenge posed by HPAI is that many strains have been found to be oseltamivir-resistant [20].

HPAI strains continue to evolve and represent a threat, as recently highlighted by a fatal case in the state of Washington, which was the first confirmed global human case of a rare H5N5 strain [21].

## 6. Closing Thoughts

Many of the viruses that might lead to a pandemic pose several challenges. For instance, recent data suggest that Chikungunya is severely underreported and often misdiagnosed as dengue fever due to a somewhat-similar symptom profile [22].

Developing vaccines—whether the more conventional forms, such as whole-virion inactivated or attenuated, subunit, and recombinant protein, or the more novel VLP, DNA vector, and mRNA-based vaccines—is one of the most important ways of preparing for potential pandemics. Finding reliable markers of immunity, weather from infection or vaccination, poses additional challenges. This was very evident during the previous pandemics—such as those caused by Influenza A(H1N1)pdm09 and, most recently, by SARS-CoV-2—when reliable correlates of protection were difficult to standardize.

Furthermore, developing novel antivirals and repurposing previously well-known medications are also essential parts of strategies for addressing potential pandemic viruses. This was made very clear during the COVID-19 pandemic, where both methods were successfully used. Discussing public health, epidemiological, pharmaceutical, immunological, and other measures that might help us avoid or at least mitigate future pandemics and thus prevent loss of life and decrease economic burdens is of utmost importance.
